# Association Between the Hemoglobin, Albumin, Lymphocyte, and Platelet (HALP) Score and Adverse Outcomes in Critically Ill Patients With Acute Myocardial Infarction: A Retrospective Study and Machine Learning Analysis

**DOI:** 10.31083/RCM43942

**Published:** 2025-08-20

**Authors:** Zhantao Cao, Ningjing Chen, Hanjing Jiang, Jian Li, Kailin Zheng, Jingting Chen, Yunsu Wang, Jun Chen

**Affiliations:** ^1^Department of Cardiology, Xiamen Hospital of Traditional Chinese Medicine, Fujian University of Traditional Chinese Medicine, 361015 Xiamen, Fujian, China; ^2^Department of Breast Surgery, Xiamen Hospital of Traditional Chinese Medicine, Fujian University of Traditional Chinese Medicine, 361015 Xiamen, Fujian, China

**Keywords:** HALP score, acute myocardial infarction, intensive care unit, MIMIC-IV database, machine learning

## Abstract

**Background:**

The hemoglobin, albumin, lymphocyte, and platelet (HALP) score represents a meaningful predictor in many cardiovascular diseases. However, the predictive utility of this score for the outcome of patients admitted to the intensive care unit (ICU) due to acute myocardial infarction (AMI) has yet to be fully elucidated.

**Methods:**

Information from the Medical Information Mart for Intensive Care (MIMIC)-IV v3.1 database was used to analyze the association between the HALP score and 90 days and 365 days all-cause mortality in critically ill patients with AMI. Patients were grouped according to the calculated HALP quartiles. Cox proportional hazards regression analysis and restricted cubic spline (RCS) analysis were performed to assess the association between the HALP score and mortality risk. A recursive algorithm identified the HALP inflection point, thus defining high and low HALP groups for the Kaplan–Meier survival analysis. Subgroup analyses analyzed the robustness across clinical strata. Furthermore, predictive models based on machine learning algorithms that included the HALP score were constructed to estimate 90 days mortality. The performance of the models was evaluated using the area under the receiver operating characteristic curve (AUC).

**Results:**

A total of 818 AMI patients were included. The analysis revealed mortality rates of 31% at 90 days and 40% at 365 days. Elevated HALP values were independently linked to a reduced risk of death. In fully adjusted models, patients in the top HALP quartile exhibited significantly lower all-cause mortality at 90 days (hazard ratio (HR) = 0.68; 95% confidence interval (CI): 0.47–0.99; *p* = 0.047) and 365 days (HR = 0.66; 95% CI: 0.47–0.90; *p* = 0.011). A nonlinear, inverse “L-shaped” association was observed, with an inflection point identified at a HALP value of 19.41. Below this value, each unit increase in the HALP score reduced mortality risk by 2.4%–2.7%. The Kaplan–Meier curves confirmed an improved survival above the threshold. Meanwhile, the subgroup analyses revealed a generally consistent association between the HALP score and mortality, except for age, where a significant interaction was observed (*p* = 0.003), indicating a stronger protective effect in older patients. Machine learning analyses supported the robustness and predictive value of the HALP score, with a maximum AUC of 0.7804.

**Conclusions:**

The HALP score is significantly associated with all-cause mortality among critically ill individuals suffering from AMI.

## 1. Introduction 

Over recent decades, cardiovascular diseases represent a primary cause of mortality worldwide. In the year 2021, these conditions were responsible for an estimated 20.5 million deaths worldwide. Of these, around 8.99 million were due to ischemic cardiovascular conditions such as acute myocardial infarction (AMI) [1, 2]. AMI is a particularly severe and frequent presentation of ischemic heart disease. The incidence of AMI increases markedly with age, affecting as many as 9.5% of individuals over the age of 60 years [3]. Critically ill patients in the intensive care unit (ICU) often exhibit a range of intricate health issues and coexisting risk factors. Studies indicate that approximately 4%–14% of ICU patients experience AMI during hospitalization [4]. Despite these observations, there is still only limited research on prognostic indicators and risk stratification in critically ill patients with AMI. It is therefore imperative to conduct additional studies allowing a deeper understanding of this high-risk cohort. Timely recognition and proper management of identified risk factors are essential for lowering the mortality rate in this patient population.

AMI involves complex immunological and inflammatory responses. Previous studies have suggested that combined biomarkers, including the Systemic Immune-Inflammation Index [5], Systemic Inflammatory Response Index [6], neutrophil-to-lymphocyte ratio (NLR) [7], Prognostic Nutritional Index [8], and Controlling Nutritional Status score [9], may have superior prognostic value for AMI compared with single inflammatory or nutritional markers alone [10].

The Hemoglobin, Albumin, Lymphocyte, and Platelet (HALP) score was first proposed as a prognostic tool for various types of cancers [11, 12, 13]. Recent studies have also demonstrated prognostic utility for HALP in various cardiovascular conditions, such as acute heart failure [14], coronary artery disease [15], patients undergoing percutaneous coronary intervention (PCI) [16], and individuals recovering from coronary artery bypass grafting (CABG) [17]. This evidence has increased the acceptance of HALP as a composite marker reflecting both systemic inflammation and nutritional state in the field of cardiovascular medicine. However, only limited research has been directed at specifically evaluating the prognostic relevance of the HALP score in individuals diagnosed with AMI. This gap is particularly pronounced for the high-risk subpopulation of critically ill patients with AMI, whose complex pathophysiology and management in ICU necessitates more precise risk stratification tools. Considering that AMI is accompanied by significant immune-inflammatory activation and nutritional disturbances [18, 19], we hypothesized that the HALP score may be a robust prognostic tool for predicting outcomes in this cohort. Consequently, the aim of our study was to evaluate the association between HALP score and all-cause mortality among critically ill patients with AMI.

## 2. Methods

### 2.1 Study Population

All patient information for this analysis was obtained from the Medical Information Mart for Intensive Care (MIMIC)-IV version 3.1 database (http://physionet.org/content/mimiciv/3.1/). The database comprises a vast collection of de-identified electronic health records on critically ill patients admitted to the ICU at Beth Israel Deaconess Medical Center during 2008–2022. It includes a wide range of patient-specific information, including demographic details, diagnostic classifications, vital parameters, laboratory findings, medication usage, and discharge status [20]. Investigator ZC obtained access to the MIMIC-IV database (ID: 14336451) after fulfilling the training requirements of the Collaborative Institutional Training Initiative (CITI) program.

**Fig. 1.**
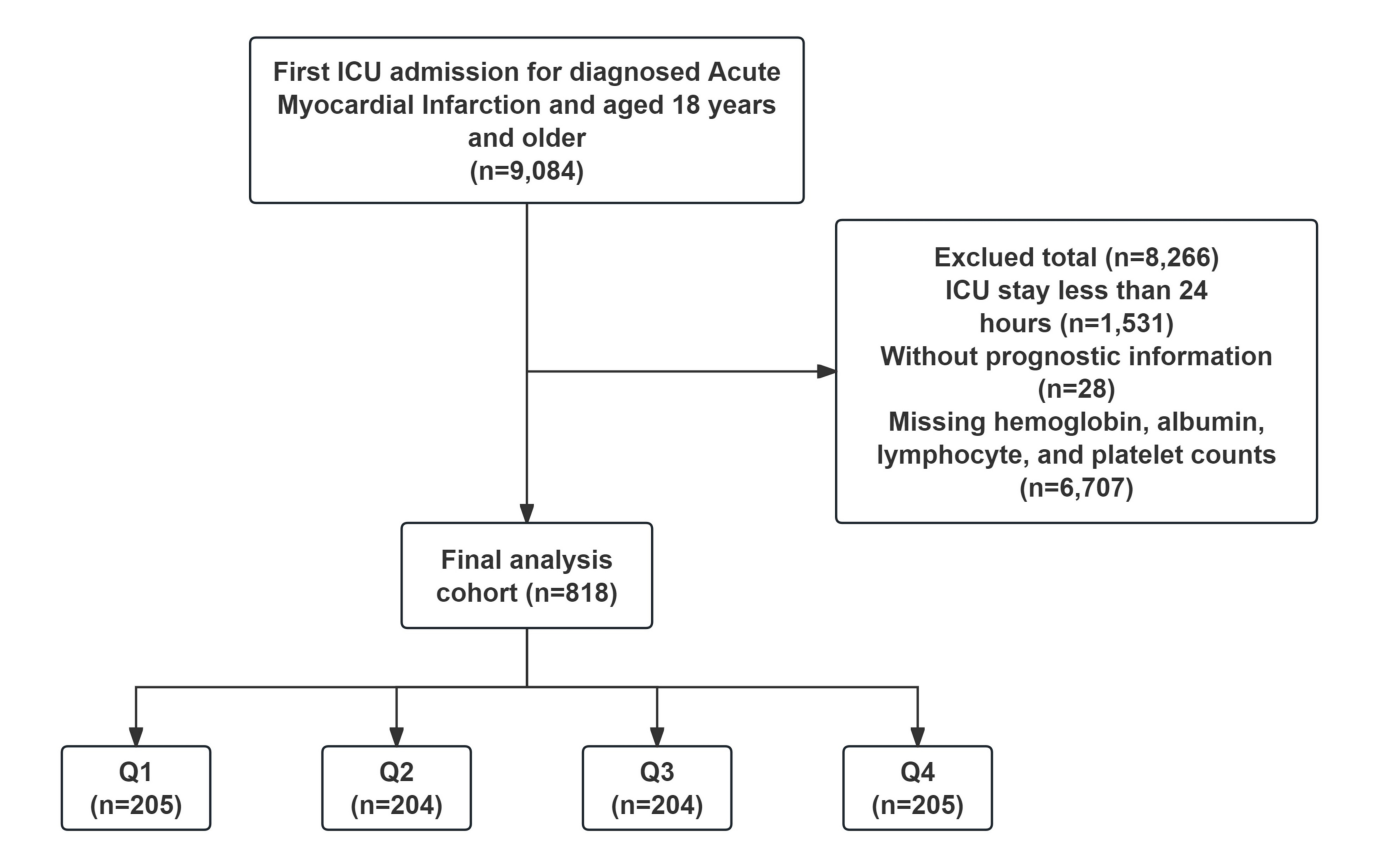
**Patient screening flow from the Medical Information Mart for Intensive Care (MIMIC)**-**IV database**. ICU, intensive care unit.

The study cohort comprised 9084 adults aged ≥18 years with a first-time ICU admission and a diagnosis of AMI (codes International Classification of Diseases-9 [ICD-9] or ICD-10). Removed from the final analysis were 1531 patients with an ICU stay of <24 h, 28 cases with no outcome data, and 6707 patients that were missing essential laboratory parameters required to compute the HALP score. A final cohort of 818 patients met the selection criteria and were divided into four groups according to the quartile distribution of their HALP score (Fig. 1).

### 2.2 Data Extraction

Using pgAdmin4 (version 8.12; pgAdmin Development Team, Chicago, IL, USA) and SQL, 7 categories of data were extracted: demographics, vital signs, laboratory indicators, underlying comorbidities, medication usage, clinical interventions, and severity scores. A full summary of all included variables is available in **Supplementary Table 1**. Only the initial lab values collected in the first 24 h following ICU admission were included in the analysis. Variables in which data was missing for >20% of cases were omitted from further analysis. For variables below this cutoff, missing values were imputed using multivariate imputation by chained equations (MICE) implemented in R (mice package, version 3.17.0, Stef van Buuren, Utrecht, Netherlands). A total of five imputations (m = 5) were conducted using a random forest (RF) algorithm to capture potential nonlinear relationships among variables. A fixed random seed (2025) was set to ensure reproducibility. The first completed dataset was used for all downstream analyses.

### 2.3 Outcomes

The main outcome assessed in this study was 90 days all-cause mortality. 365 days all-cause mortality was the secondary outcome.

### 2.4 Calculation of HALP Score

The HALP score was calculated according to the following formula [21]: hemoglobin (g/L) × albumin (g/L) × lymphocyte count (10^9^/L) / platelet count (10^9^/L). Baseline values for hemoglobin and albumin in the MIMIC-IV database were recorded in grams per deciliter (g/dL). These values were converted to grams per liter (g/L) prior to the calculation by multiplying by 10.

### 2.5 Statistical Analysis

Normality testing of all continuous variables indicated they did not follow a normal distribution. Therefore, they were presented as medians and interquartile ranges (IQRs), and the Kruskal–Wallis rank-sum test was used for comparisons between groups. Categorical data were summarized by frequencies (percentages), with group differences assessed via Pearson’s chi-square test.

All variables incorporated into the model were examined for potential multicollinearity. To reduce multicollinearity, variables exhibiting a variance inflation factor of ≥5 were removed from the model (**Supplementary Table 2**). Cox proportional hazards regression was used to determine the association between the HALP score and risk of mortality. The selection of covariates for the final models was informed by a combination of least absolute shrinkage and selection operator (LASSO) regression results and clinical judgment. Model 1 comprised only the HALP score. Model 2 was additionally adjusted for both age and gender. Model 3 included additional adjustments for race, respiratory rate (RR), systolic/diastolic blood pressure (SBP/DBP), peripheral capillary oxygen saturation (SPO_2_), carbon dioxide partial pressure (PCO_2_), white blood cell count (WBC), serum potassium, sodium, glucose (GLU), anion gap, lactate, partial thromboplastin time (PTT), atrial fibrillation (AF), cancer (CA), chronic obstructive pulmonary disease (COPD), chronic kidney disease (CKD), diabetes, hypertension, congestive heart failure (CHF), stroke, clopidogrel use, beta-blockers, statins, invasive mechanical ventilation (MV), and noninvasive MV.

To explore potential nonlinear trends, restricted cubic spline (RCS) analysis was employed to examine the link between the HALP score and all-cause mortality. When a statistically significant nonlinear association was found, a recursive algorithm was used to determine the inflection point for the HALP score in relation to 90 days and 365 days mortality. For a deeper analysis of the link between the HALP score and mortality, segmented Cox regression models were fitted separately for the ranges below and above the identified turning point. Based on this inflection point, patients were then divided into low- and high-HALP groups. Kaplan–Meier (KM) survival analysis was conducted to compare the occurrence of outcomes between these groups.

In addition, subgroup evaluations were carried out among populations defined according to age, gender, AF, hypertension, CHF, and diabetes. Interaction effects between the HALP score and each stratification variable were evaluated through likelihood ratio testing. All statistical computations were performed using R software (version 4.4.3; R Foundation for Statistical Computing, Vienna, Austria), with statistical significance set at a two-sided *p*-value of <0.05.

### 2.6 Construction and Assessment of the Prognostic Models

The dataset was randomly partitioned into a training cohort (70% of data) and a validation cohort (30% of data). In the training cohort, feature selection was performed using LASSO regression, with five-fold cross-validation to determine the optimal λ parameter. The variables identified were subsequently employed to construct a series of machine learning models for the prediction of 90 days mortality in AMI patients.

To optimize the performance of each model, hyperparameters were systematically tuned using a grid search strategy combined with five-fold cross-validation. The basis for selecting the final hyperparameters was to maximize the mean area under the receiver operating characteristic curve (AUC) during the cross-validation process. The specific hyperparameter tuning ranges and the final selected values for each model are detailed in **Supplementary Table 3**. The developed models included support vector machine, elastic net (ENet), decision tree, Light Gradient Boosting Machine (LightGBM), ridge regression, multilayer perceptron (MLP), RF, k-nearest neighbors, extreme gradient boosting (XGBoost) algorithms, and Stacking ensemble algorithms. Discrimination was measured by calculating the AUC.

Furthermore, the clinical utility of various models was assessed using decision curve analysis (DCA). Calibration curves were also generated to evaluate the concordance between predicted probabilities and observed results. To enhance model interpretability and facilitate clinical translation, SHapley Additive exPlanations (SHAP) were utilized to interpret the predictions of the optimal model.

## 3. Results

### 3.1 Baseline Characteristics

A final cohort of 818 patients with AMI met the criteria for inclusion in this analysis. The median age of participants in the study cohort was 71 years (IQR: 62–80), with males accounting for 62% of the population. Participants were allocated to one of four groups according to the quartile distribution of their HALP scores upon ICU admission: Q1 (HALP <9.7), Q2 (9.7 ≤ HALP < 19.71), Q3 (19.71 ≤ HALP < 34.47), and Q4 (HALP ≥34.47). The baseline features of each subgroup are summarized in Table 1. To address potential selection bias stemming from the exclusion of 6707 patients who were missing the necessary parameters to calculate the HALP score, their baseline characteristics were compared against those of the 818 patients included in the final analysis (**Supplementary Table 4**). The comparison revealed significant differences between the two groups. Notably, the included cohort presented with a more severe clinical profile, as evidenced by higher rates of sepsis (73% vs. 58%, *p* < 0.001), higher severity scores (median simplified acute physiology score II [SAPS-II]: 41 vs. 37, *p* < 0.001; median sequential organ failure assessment [SOFA]: 6 vs. 4, *p* < 0.001), and a greater need for continuous renal replacement therapy (9.5% vs. 3.5%, *p* < 0.001). Crucially, the included patients experienced significantly higher all-cause mortality at both 90 days (31% vs. 18%, *p* < 0.001) and 365 days (40% vs. 26%, *p* < 0.001) compared to the excluded group.

**Table 1.** **Characteristics and outcomes of participants categorized by HALP score**.

Characteristic		Overall (n = 818)	Q1 (HALP <9.7, n = 205)	Q2 (9.7 ≤ HALP < 19.71, n = 204)	Q3 (19.71 ≤ HALP < 34.47, n = 204)	Q4 (HALP ≥34.47, n = 205)	*p*-value
Age (years)		71 (62, 80)	71 (62, 80)	72 (62, 81)	70 (61, 80)	70 (62, 78)	0.449
Gender, n (%)							0.212
	Female	314 (38%)	81 (40%)	79 (39%)	87 (43%)	67 (33%)	
	Male	504 (62%)	124 (60%)	125 (61%)	117 (57%)	138 (67%)	
Race, n (%)							0.486
	Black	71 (8.7%)	14 (6.8%)	20 (9.8%)	23 (11%)	14 (6.8%)	
	White	475 (58%)	117 (57%)	116 (57%)	122 (60%)	120 (59%)	
	Others	272 (33%)	74 (36%)	68 (33%)	59 (29%)	71 (35%)	
Heart rate (bpm)		87 (75, 101)	92 (78, 104)	88 (78, 106)	87 (75, 98)	83 (73, 96)	0.002
RR (bpm)		20 (16, 25)	21 (17, 26)	20 (17, 25)	20 (17, 25)	20 (16, 23)	0.008
SBP (mmHg)		118 (104, 137)	120 (104, 138)	115 (103, 134)	120 (104, 138)	120 (105, 138)	0.527
DBP (mmHg)		69 (59, 81)	68 (59, 81)	68 (58, 82)	70 (59, 79)	70 (59, 82)	0.912
SPO_2_ (%)		97 (94, 100)	97 (94, 100)	97 (93, 99)	97 (94, 99)	98 (95, 100)	0.090
Hemoglobin (g/dL)		10.65 (8.80, 12.70)	9.10 (7.80, 11.00)	10.45 (9.00, 11.85)	11.40 (9.70, 13.55)	11.70 (9.50, 13.50)	<0.001
Albumin (g/dL)		3.20 (2.80, 3.60)	2.90 (2.50, 3.30)	3.10 (2.70, 3.50)	3.40 (2.90, 3.70)	3.50 (3.00, 3.80)	<0.001
Lymph (10^9^/L)		1.07 (0.65, 1.70)	0.44 (0.29, 0.74)	0.87 (0.69, 1.20)	1.30 (0.98, 1.71)	1.92 (1.53, 2.71)	<0.001
Platelet (10^9^/L)		198 (141, 255)	226 (166, 317)	208 (167, 263)	197 (144, 243)	155 (99, 214)	<0.001
INR		1.30 (1.10, 1.50)	1.30 (1.20, 1.60)	1.30 (1.20, 1.55)	1.25 (1.10, 1.50)	1.20 (1.10, 1.60)	0.106
PH		7.36 (7.29, 7.42)	7.35 (7.28, 7.41)	7.37 (7.29, 7.42)	7.37 (7.28, 7.42)	7.37 (7.32, 7.42)	0.312
PTT (S)		34 (28, 51)	31 (27, 40)	34 (28, 47)	36 (28, 58)	35 (29, 66)	<0.001
WBC (10^9^/L)		13 (9, 17)	12 (9, 17)	13 (10, 17)	13 (9, 17)	12 (9, 18)	0.161
PCO_2_ (mmHg)		41 (35, 47)	41 (35, 49)	40 (35, 48)	40 (35, 46)	41 (36, 45)	0.768
Cr (mg/dL)		1.30 (0.90, 2.10)	1.60 (1.00, 3.10)	1.40 (0.90, 2.40)	1.30 (0.90, 1.80)	1.10 (0.80, 1.60)	<0.001
Potassium (mmol/L)		4.30 (3.90, 4.70)	4.30 (3.90, 4.90)	4.30 (3.90, 4.70)	4.30 (3.90, 4.80)	4.10 (3.80, 4.50)	0.036
Sodium (mmol/L)		138 (136, 141)	138 (134, 141)	138 (136, 141)	139 (136, 141)	139 (136, 141)	0.017
BUN (mg/dL)		26 (16, 46)	36 (20, 59)	29 (18, 46)	24 (16, 42)	20 (14, 36)	<0.001
Lactate (mmol/L)		1.90 (1.30, 3.00)	1.90 (1.30, 2.90)	1.80 (1.30, 3.10)	1.95 (1.30, 3.00)	1.90 (1.30, 3.20)	0.712
GLU (mg/dL)		147 (113, 207)	154 (111, 209)	142 (116, 204)	151 (120, 206)	140 (105, 196)	0.449
PO_2_ (mmHg)		66 (41, 122)	55 (38, 93)	66 (41, 115)	64 (41, 120)	86 (49, 234)	<0.001
Anion gap (mmol/L)		15 (13, 18)	16 (13, 19)	15 (13, 18)	15 (13, 18)	15 (12, 17)	0.197
Neuts (10^9^/L)		10 (7, 15)	10 (7, 15)	11 (7, 15)	10 (8, 15)	10 (6, 14)	0.295
AF, n (%)		368 (45%)	103 (50%)	92 (45%)	89 (44%)	84 (41%)	0.287
CA, n (%)		167 (20%)	44 (21%)	45 (22%)	37 (18%)	41 (20%)	0.765
CKD, n (%)		320 (39%)	95 (46%)	93 (46%)	68 (33%)	64 (31%)	<0.001
CHF, n (%)		506 (62%)	131 (64%)	135 (66%)	119 (58%)	121 (59%)	0.290
COPD, n (%)		180 (22%)	59 (29%)	43 (21%)	36 (18%)	42 (20%)	0.044
Diabetes, n (%)		360 (44%)	94 (46%)	82 (40%)	89 (44%)	95 (46%)	0.583
Hypertension, n (%)		370 (45%)	100 (49%)	70 (34%)	92 (45%)	108 (53%)	0.001
Sepsis, n (%)		596 (73%)	171 (83%)	156 (76%)	129 (63%)	140 (68%)	<0.001
Stroke, n (%)		126 (15%)	27 (13%)	29 (14%)	35 (17%)	35 (17%)	0.588
Aspirin, n (%)		539 (66%)	111 (54%)	129 (63%)	138 (68%)	161 (79%)	<0.001
Beta-blockers, n (%)		481 (59%)	122 (60%)	104 (51%)	123 (60%)	132 (64%)	0.046
Clopidogrel, n (%)		156 (19%)	37 (18%)	50 (25%)	37 (18%)	32 (16%)	0.124
Statin, n (%)		540 (66%)	111 (54%)	132 (65%)	143 (70%)	154 (75%)	<0.001
CRRT, n (%)		78 (9.5%)	24 (12%)	23 (11%)	14 (6.9%)	17 (8.3%)	0.273
Invasive MV, n (%)		396 (48%)	96 (47%)	101 (50%)	101 (50%)	98 (48%)	0.933
Noninvasive MV, n (%)		21 (2.6%)	5 (2.4%)	10 (4.9%)	4 (2.0%)	2 (1.0%)	0.077
APS-III		48 (35, 65)	55 (42, 69)	50 (38, 61)	43 (32, 58)	43 (32, 63)	<0.001
CCI		6 (5, 9)	7 (5, 9)	7 (5, 9)	6 (5, 8)	6 (4, 8)	<0.001
GCS		15 (14, 15)	15 (14, 15)	15 (14, 15)	15 (14, 15)	15 (14, 15)	0.253
SAPS-II		41 (31, 52)	44 (34, 54)	42 (34, 50)	39 (29, 50)	40 (31, 52)	0.004
SOFA		6 (3, 10)	7 (4, 10)	6 (4, 9)	5 (3, 10)	7 (3, 10)	0.229
90 days mortality, n (%)		250 (31%)	75 (37%)	70 (34%)	53 (26%)	52 (25%)	0.024
365 days mortality, n (%)		330 (40%)	99 (48%)	87 (43%)	75 (37%)	69 (34%)	0.013

HALP, Hemoglobin, Albumin, Lymphocyte, and Platelet; RR, respiratory rate; SBP, systolic blood pressure; DBP, diastolic blood pressure; SPO_2_, peripheral capillary oxygen saturation; Hb, hemoglobin; INR, international normalized ratio; PH, potential of hydrogen; PTT, partial thromboplastin time; WBC, white blood cell count; PCO_2_, partial pressure of carbon dioxide; Cr, creatinine; BUN, blood urea nitrogen; GLU, glucose; PO_2_, partial pressure of oxygen; AF, atrial fibrillation; CA, cancer; CKD, chronic kidney disease; CHF, congestive heart failure; COPD, chronic obstructive pulmonary disease; CRRT, continuous renal replacement therapy; MV, mechanical ventilation; APS-III, acute physiology score III; CCI, Charlson comorbidity index; GCS, Glasgow coma scale; SAPS-II, simplified acute physiology score II; SOFA, sequential organ failure assessment.

Patients in the highest HALP quartile (Q4) were generally younger and included a greater proportion of males compared to the lowest quartile (Q1). The Q4 group also showed higher levels of albumin, hemoglobin, lymphocyte count, sodium, and PTT. In contrast, patients in Q4 had lower heart rate, RR, platelet count, international normalized ratio (INR), creatinine (Cr), blood urea nitrogen (BUN), potassium, GLU, anion gap, and Acute Physiology Score III (APS-III). Additionally, the prevalence of AF, CA, CHF, and sepsis was lower in Q4, along with less use of beta-blockers and clopidogrel.

In comparison with the other quartiles, the Q4 group had a lower mortality rate at all evaluated time points. The 90 days mortality rates were 37%, 34%, 26%, and 25% for Q1 to Q4, respectively (*p* = 0.024), while the mortality rates at 365 days were 48%, 43%, 37%, and 34%, respectively (*p* = 0.013).

### 3.2 Relationship Between HALP Score and Clinical Outcomes

The association between the HALP score and mortality risk was investigated using Cox proportional hazards regression analysis, as shown in Table 2. In the unadjusted analysis (Model 1), the highest HALP quartile (Q4) was associated with a significantly reduced risk of 90 days mortality relative to the lowest quartile (Q1), with a hazard ratio (HR) of 0.66 and 95% confidence interval (CI) of 0.46–0.94 (*p* = 0.020). The association with reduced risk persisted following adjustment for age and gender in Model 2 (HR = 0.65, 95% CI: 0.46–0.92; *p* = 0.016). The reduced risk was still apparent following complete adjustment for comorbidities, laboratory findings, and medication use in Model 3 (HR = 0.68, 95% CI: 0.47–0.99; *p* = 0.047). A very similar association was also evident for 365 days mortality (Table 2). The trend analysis demonstrated a significant dose-response pattern, where higher HALP quartiles were linked with a stepwise decrease in all-cause mortality risk (all *p* for trend <0.05). These results indicate that an elevated HALP score is independently associated with a reduced risk of mortality.

**Table 2.** **Association between HALP score and all-cause mortality at 90 days and 365 days**.

Variables		Model 1		Model 2		Model 3	
		HR (95% CI)	*p*-value	HR (95% CI)	*p*-value	HR (95% CI)	*p*-value
90 days mortality							
HALP quartile							
	Q1	1.00 (Reference)		1.00 (Reference)		1.00 (Reference)	
	Q2	0.95 (0.68∼1.13)	0.737	0.91 (0.66∼1.26)	0.567	0.84 (0.60∼1.17)	0.298
	Q3	0.69 (0.48∼0.97)	0.035	0.67 (0.49∼0.98)	0.040	0.66 (0.46∼0.96)	0.029
	Q4	0.66 (0.46∼0.94)	0.020	0.65 (0.46∼0.92)	0.016	0.68 (0.47∼0.99)	0.047
*p* for trend			0.005		0.006		0.022
365 days mortality							
HALP quartile							
	Q1	1.00 (Reference)		1.00 (Reference)		1.00 (Reference)	
	Q2	0.87 (0.65∼1.16)	0.341	0.83 (0.63∼1.11)	0.218	0.80 (0.60∼1.08)	0.152
	Q3	0.71 (0.52∼0.95)	0.023	0.70 (0.52∼0.95)	0.022	0.69 (0.50∼0.94)	0.020
	Q4	0.63 (0.47∼0.86)	0.004	0.62 (0.46∼0.85)	0.003	0.66 (0.47∼0.90)	0.011
*p* for trend			<0.001		<0.001		0.006

Model 1: Crude. Model 2: Adjusted for Age and Gender. Model 3: Adjusted for Age, Gender, Race, RR, SBP, DBP, SPO_2_, WBC, PCO_2_, Potassium, Sodium, GLU, Anion gap, lactate, PTT, AF, CA, CKD, CHF, COPD, Diabetes, Hypertension, Stroke, Clopidogrel, Beta-blockers, Statin, Invasive MV, and Noninvasive MV. HR, Hazard Ratio; CI, Confidence Interval.

### 3.3 Detection of Nonlinear Relationship

**Fig. 2.**
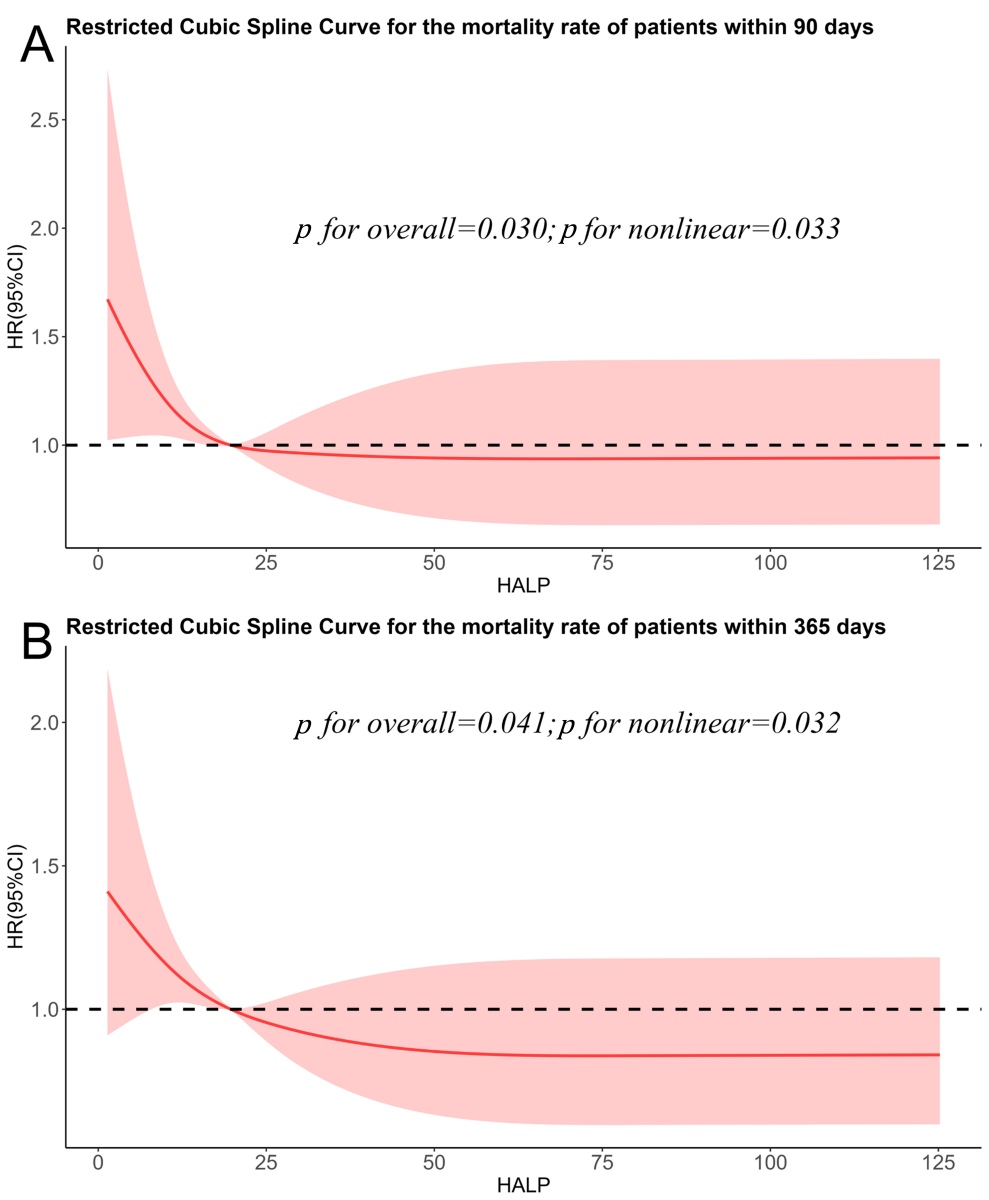
**Restricted cubic spline (RCS) analysis of the association between Hemoglobin, Albumin, Lymphocyte, and Platelet (HALP) score and all-cause mortality at 90 days (A) and 365 days (B)**.

The RCS analysis suggested a possible nonlinear relationship linking the HALP score to all-cause mortality at each time point (both *p* for nonlinear <0.05). Specifically, the association exhibited an inverse L-shaped pattern, indicating a sharp decline in mortality risk with increasing HALP scores up to a certain point, beyond which the effect plateaued (Fig. 2).

To further explore this nonlinear relationship, we applied both conventional and two-piece Cox proportional hazards models, as shown in Table 3. Log-likelihood ratio tests confirmed a superior statistical fit for the two-piece model (*p* < 0.05 for all comparisons). For both 90 days and 365 days all-cause mortality, the analysis identified a HALP score of 19.41 as the inflection point. Below the inflection point (HALP score ≤19.41), each one-unit rise was associated with a 2.7% reduction in 90 days mortality risk (HR = 0.973, 95% CI: 0.952–0.994, *p* = 0.012) and a 2.4% reduction in 365 days mortality risk (HR = 0.976, 95% CI: 0.957–0.994, *p* = 0.011). In contrast, when the HALP score exceeded 19.41, it was no longer significantly associated with mortality at either time point (*p* > 0.05).

**Table 3.** **Threshold effect analysis of HALP score on all-cause mortality**.

90 days mortality		HR (95% CI), *p*-value
Inflection point		19.41
Fitting model by two-piecewise linear regression		
	HALP ≤19.41	0.973 (0.952∼0.994), 0.012
	HALP >19.41	1.000 (1.000∼1.000), 0.161
	*p* for Log-likelihood ratio	0.013
365 days mortality		HR (95% CI), *p*-value
Inflection point		19.41
Fitting model by two-piecewise linear regression		
	HALP ≤19.41	0.976 (0.957∼0.994), 0.011
	HALP >19.41	1.000 (1.000∼1.000), 0.260
	*p* for Log-likelihood ratio	0.012

HALP, Hemoglobin, Albumin, Lymphocyte, and Platelet; HR, Hazard Ratio; CI, Confidence Interval.

### 3.4 KM Survival Curves

**Fig. 3.**
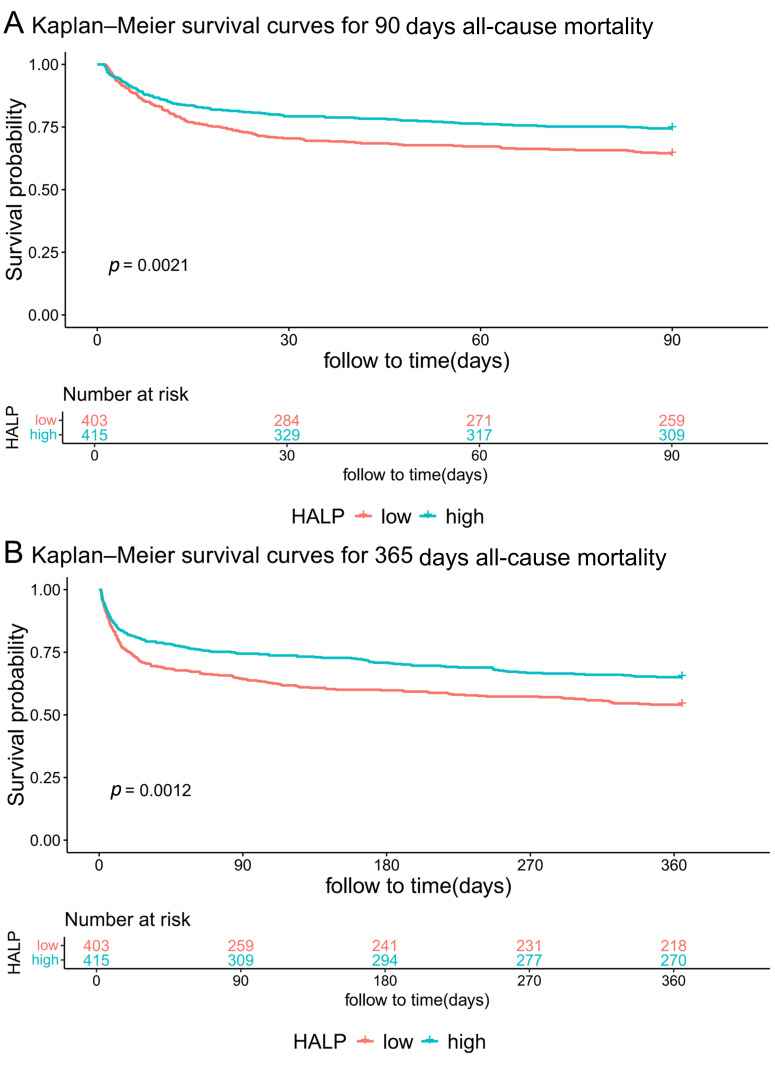
**Kaplan–Meier survival curves for all-cause mortality at 90 days (A) and 365 days (B) of patients with high and low Hemoglobin, Albumin, Lymphocyte, and Platelet (HALP) score**.

For the KM survival analysis, patients were stratified into high and low HALP groups using the inflection point of 19.41 as the threshold (Fig. 3). The low HALP score group had significantly worse 90 days survival relative to the high HALP group (*p* = 0.002). A comparable and statistically significant result was also observed for 365 days all-cause mortality.

### 3.5 Subgroup Analysis

**Fig. 4.**
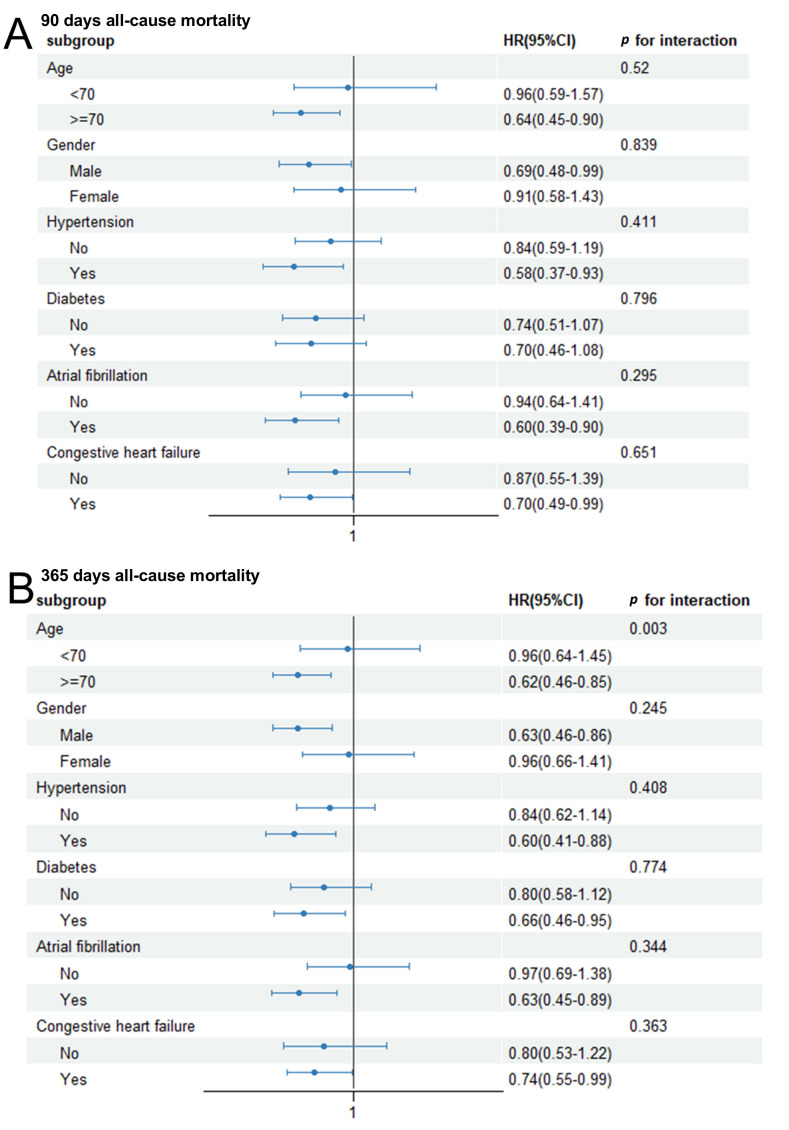
**Subgroup analysis for the association between 90 days (A) or 365 days (B) all-cause mortality and Hemoglobin, Albumin, Lymphocyte, and Platelet (HALP) score**.

We next performed subgroup analyses to determine whether the link between the HALP score and 90 days and 365 days all-cause mortality was consistent across different clinical subgroups. These analyses stratified patients by age, gender, hypertension, diabetes, AF, and CHF (Fig. 4). A significantly lower risk of 90 days mortality was associated with higher HALP scores in individuals aged ≥70 years (HR = 0.64), males (HR = 0.69), and those with hypertension (HR = 0.58), atrial fibrillation (HR = 0.60), or congestive heart failure (HR = 0.70). The association was not significant at this time point in patients with diabetes. The protective association with higher HALP scores was even more widespread for 365 days mortality. It remained statistically significant in all of the aforementioned subgroups, while also being significant in patients with diabetes (HR = 0.66). Interaction analysis revealed a significant effect modification by age for 365 days mortality (*p* for interaction = 0.003). Specifically, higher HALP scores were strongly linked to reduced mortality in patients aged ≥70 years (HR = 0.62; 95% CI: 0.46–0.85), but this association was not significant in those aged <70 years (HR = 0.96; 95% CI: 0.64–1.45). No other significant interactions were observed, indicating the prognostic utility of HALP is particularly evident in elderly patients.

### 3.6 Contribution and Interaction of HALP Components

We carried out two additional analyses to determine if the prognostic value of HALP is disproportionately driven by any single component.

First, a dominance analysis was performed to evaluate the comparative importance of each HALP component. As shown in **Supplementary Table 5**, albumin accounted for 77.8% of the overall predictive contribution, followed by hemoglobin (13.5%), platelets (7.9%), and lymphocytes (0.8%). This result indicates that albumin is the primary contributor to the prognostic value of the HALP score. Second, to test for potential synergistic or antagonistic effects, interaction terms between HALP components were incorporated into a multivariable Cox regression model. None of the interaction terms (e.g., albumin × hemoglobin) achieved statistical significance (all *p* > 0.05), indicating that each component contributes independently to risk prediction (**Supplementary Table 6**). These analyses support the internal validity and stability of HALP as a composite biomarker in critically ill AMI patients.

### 3.7 Feature Selection

**Fig. 5.**
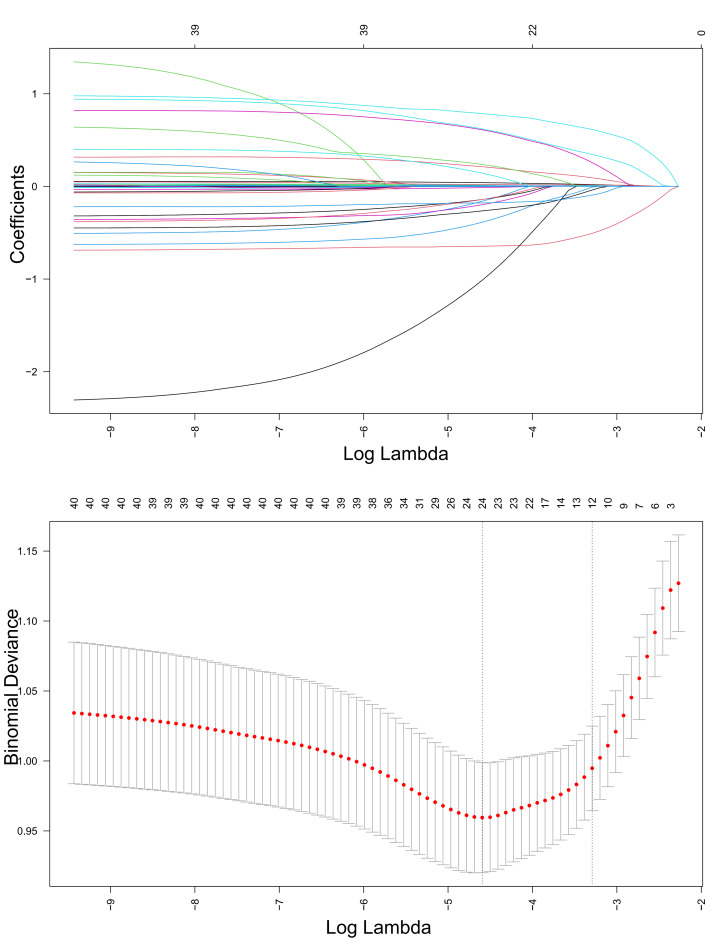
**Least absolute shrinkage and selection operator (LASSO) regression-based screening of variables**.

As shown in Fig. 5, LASSO regression was applied to the training cohort to identify the most relevant predictive features. During model construction, five-fold cross-validation was utilized to determine the optimal penalty parameter (λ). The λ value associated with the lowest cross-validation error (lambda.min) was chosen to optimize the trade-off between model accuracy and feature sparsity. At the lambda.min point, a total of 24 variables were identified as the most predictive of all-cause mortality and were used to construct the final analysis model: age, gender, race, RR, SPO_2_, HALP score, INR, PTT, sodium, BUN, lactate, anion gap, AF, CA, CKD, CHF, hypertension, sepsis, aspirin, beta-blockers, statin, renal replacement therapy, invasive MV, and noninvasive MV.

### 3.8 Construction and Validation of Prognostic Models 

**Fig. 6.**
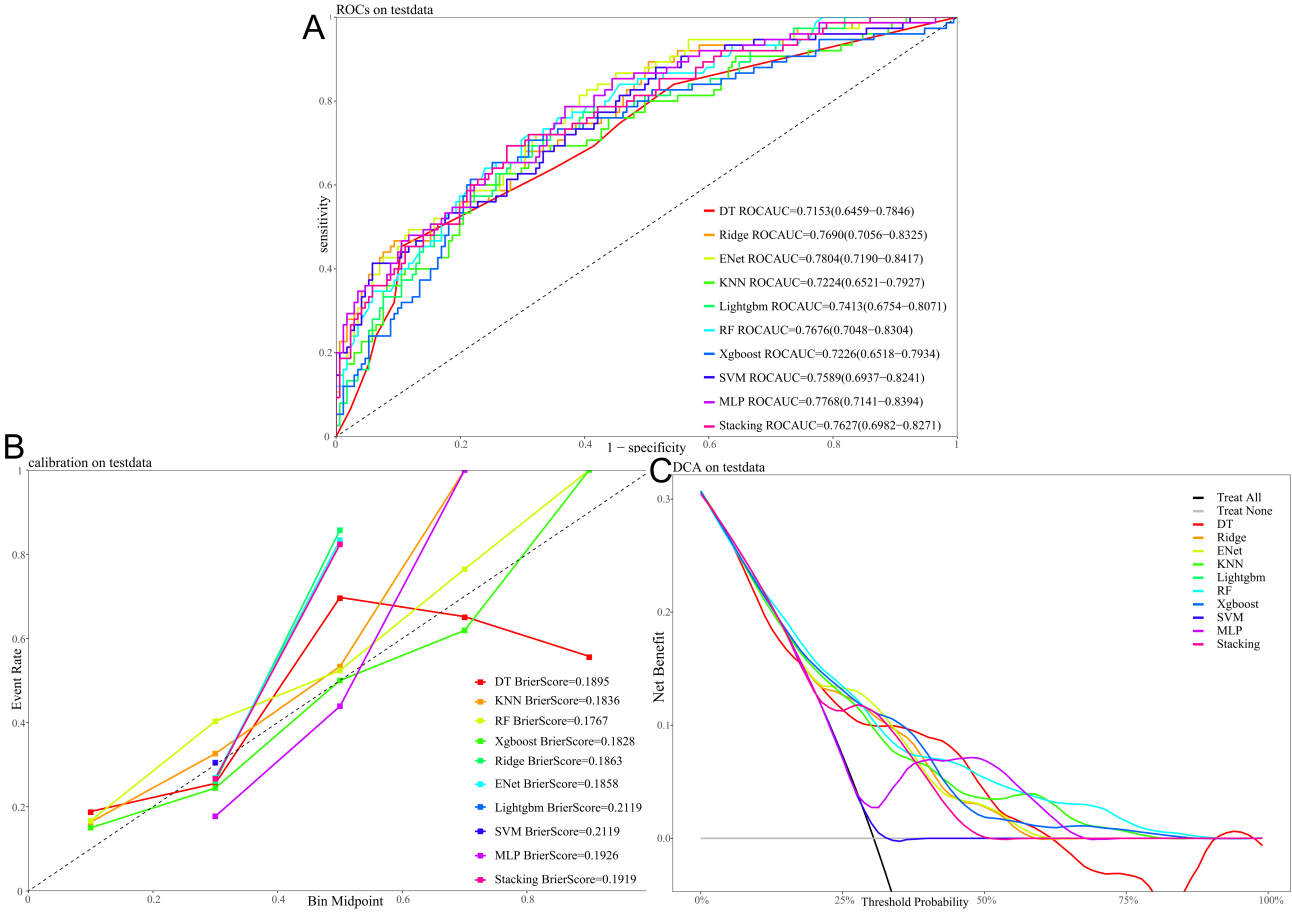
**Performance and clinical utility of different machine learning models for predicting 90 days all-cause mortality with the test dataset**. (A) Receiver operating characteristic (ROC) curves, with the area under the curve (AUC) value for each model. (B) Calibration curves, with the Brier score indicating the calibration performance for each model. (C) Decision curve analysis (DCA), showing the net benefit of using each model across a range of threshold probabilities.

The receiver operating characteristi (ROC) curves for different machine learning algorithms evaluated on the test dataset are shown in Fig. 6A, with their predictive performance assessed by the AUC. Ranked from highest to lowest AUC, the models performed as follows: ENet = 0.7804, MLP = 0.7768, ridge regression = 0.7690, RF = 0.7676, and Stacking = 0.7627. These results indicate that ENet, MLP, and ridge regression showed relatively superior predictive performance. Fig. 6B presents the calibration curves for each model on the test set. Among them, RF and XGBoost demonstrated the closest alignment with the ideal reference line and achieved the lowest Brier scores (0.1767 and 0.1828, respectively), indicating better predictive consistency and calibration. Fig. 6C illustrates the results of DCA for all models. Across a range of threshold probabilities, each model provided a clear net clinical benefit over the “treat-all” and “treat-none” strategies, further supporting the potential clinical utility and value of these predictive models.

To assess whether our ENet model offers an improvement over existing risk stratification tools, we compared its performance against models based on the Charlson Comorbidity Index (CCI) and Glasgow coma scale (GCS) alone. As shown in **Supplementary Table 7**, the addition of HALP and other variables to our ENet model led to significant improvements in both net reclassification and discrimination in the validation set. A significant improvement was observed compared to the CCI model (NRI = 0.445, *p* < 0.001; IDI = 0.079, *p* = 0.005), and an even greater improvement compared to the GCS model (NRI = 0.924, *p* < 0.001; IDI = 0.156, *p* < 0.001). Furthermore, comparative DCA indicates the ENet model offers a superior net benefit across a wide range of threshold probabilities (**Supplementary Fig. 1**), indicating superior clinical utility over the standalone scores.

### 3.9 Model Interpretability and Clinical Applicability

To enhance the clinical applicability of our best-performing model (ENet), we utilized SHAP to interpret the model’s predictions at both the global and individual levels. The SHAP summary figure (**Supplementary Fig. 2**) depicts the relative impact of each variable on the prediction of mortality. Variables such as admission age, BUN, lactate, CRRT use, and HALP had the highest impact on the model output. Of note, elevated HALP scores demonstrated a consistent association with a lower predicted risk, further supporting its inverse relationship with mortality. This interpretation was based on the ENet model, which achieved the highest AUC among all the machine learning algorithms evaluated in this study. The global importance ranking based on mean absolute SHAP values is shown in **Supplementary Fig. 3**. HALP ranked among the top predictive features, thus confirming its clinical value beyond conventional predictors. Such explainability visualizations can assist clinicians in understanding the relative importance of different risk factors, as well as enhancing trust in machine learning-driven decision support tools. Ultimately, the integration of HALP into a transparent, interpretable model framework may facilitate risk stratification and individualized treatment planning in ICU patients with AMI.

## 4. Discussion

In this study, we conducted a comprehensive examination of the link between HALP score and all-cause mortality in critically ill AMI patients. We found a significant association between higher HALP score and reduced risk of mortality at both 90 days and 365 days follow-up periods. Multivariable Cox regression models revealed that individuals with HALP scores in the upper quartile (Q4) had a significantly lower risk of death compared to those in Q1, with an HR of 0.68 for 90 days mortality and 0.66 for 365 days mortality (both *p* < 0.05). Threshold effect analyses were performed using both standard and two-piece Cox models, with an inflection point identified at a HALP score of 19.41 for both time points. Below this identified threshold, each one-point rise in HALP score corresponded to a 2.4%–2.7% decrease in mortality risk. However, no significant relationship was observed when the HALP score exceeded the cutoff. Additionally, machine learning models incorporating the HALP score and other clinical variables demonstrated strong predictive performance for 90 days mortality, with the highest AUC reaching 0.78. Furthermore, our additional analyses demonstrated that a model incorporating the HALP score provides significant incremental value in risk prediction and clinical utility over established scores like the CCI and GCS, as evidenced by NRI and DCA results. These results highlight the value of the HALP score as a predictive marker and its applicability in developing targeted interventions to reduce mortality in critically ill AMI patients.

Immune-inflammatory mechanisms are pivotal in driving the progression of AMI and determining its prognosis. Following the onset of AMI, a robust immune-inflammatory response is triggered, resulting in substantial release of damage-associated molecular patterns. These facilitate the recruitment and infiltration of neutrophils, monocytes, and macrophages into the infarcted myocardium [22, 23]. During this phase, neutrophils exacerbate the local myocardial injury by releasing proteolytic enzymes and reactive oxygen species via degranulation, thereby expanding the infarct size and initiating maladaptive left ventricular remodeling [24]. Macrophages further amplify local inflammation, thereby exacerbating ventricular dilatation and dysfunction [25]. Concurrently, hospitalized AMI patients frequently exhibit nutritional deficits, such as hypoalbuminemia and anemia, which impair immune function, reduce resistance to inflammatory damage, and diminish the capacity for tissue regeneration [26]. Studies have demonstrated that malnutrition significantly increases mortality in AMI patients and is an independent predictor of poor outcomes [27]. Thus, the exaggerated immune-inflammatory response following AMI directly exacerbates myocardial injury and adverse remodeling, while concurrent nutritional deficiencies weaken the body’s immune defence and repair mechanisms. Together, these synergistic effects contribute substantially to poor patient outcomes.

The HALP score functions as a composite biomarker that simultaneously captures both immune-inflammatory activity and nutritional condition. Our findings demonstrate a nonlinear relationship of the HALP score with mortality in critically ill AMI patients. This was identified as an inverse “L-shaped” curve by RCS analysis. A significant inflection point was detected at a HALP score of 19.41, below which the risk of mortality declined sharply with each unit increase in HALP. After this threshold, the risk of mortality stabilized. The threshold effect provides a clinically actionable cutoff, allowing stratification of ICU patients into low- and high-risk groups at the time of admission. Such early risk stratification can facilitate timely nutritional support, anti-inflammatory interventions, and intensive monitoring. The observed nonlinear relationship underscores the combined impact of immune-inflammatory responses and nutritional status on patient prognosis. Firstly, a lower HALP score suggests dual impairment of immune function and nutritional status. Lymphopenia diminishes the body’s capacity to effectively regulate and suppress inflammation. Previous studies have indicated that AMI patients with lower lymphocyte counts and higher NLR have a significantly increased risk of long-term mortality. Furthermore, impaired peripheral T-lymphocyte function has been shown to exacerbate myocardial ischemia-reperfusion injury [28, 29]. Concurrently, platelets not only contribute to coronary artery and microvascular thrombosis during AMI, but also exacerbate inflammation and reperfusion injury. This inflammatory-thrombotic interaction is recognized as a critical contributor to adverse outcomes in AMI [30, 31]. Increased platelet levels typically indicate a cytokine-driven acute-phase response that promotes inflammation, most notably interleukin-6, leading to a hypercoagulable and pro-inflammatory state [32]. Compared to patients with moderate platelet counts (250–349 K/µL), those with higher counts (≥350 K/µL) were reported to show increased overall mortality following AMI [33]. Secondly, hypoalbuminemia, as included in the HALP score, not only indicates malnutrition but also serves as a marker of increased inflammation and oxidative stress [34]. Clinical evidence consistently demonstrates that hypoalbuminemia independently predicts adverse outcomes following AMI [35]. To illustrate this, a cohort study of 7192 patients with acute coronary syndrome (ACS) found that individuals with a serum albumin level <3.5 g/dL at admission had significantly higher rates of both in-hospital death and heart failure [36]. Moreover, a lower HALP score typically coexists with reduced hemoglobin levels (anemia), directly impairing oxygen transport and tissue perfusion. Anemic states intensify myocardial hypoxia, potentially enlarging the infarct size and precipitating cardiac dysfunction. Severe anemia (hemoglobin <9 g/dL) has been shown to significantly increase short-term mortality among AMI patients, and a hemoglobin level of <9 g/dL is associated with an approximately 50% increase in the 120 days mortality risk [37]. At a mechanistic level, the robust prognostic power of the HALP score in critically ill AMI patients likely stems from the synergistic interplay among its components, which collectively reinforce a vicious cycle of inflammation, hypoxia, and impaired healing. For example, anemia-induced hypoxia can exacerbate inflammatory signaling via HIF-1α pathways [38, 39], while inflammation in turn suppresses erythropoiesis, further worsening anemia [40]. Concurrently, hypoalbuminemia not only signals depleted nutritional reserves, but also weakens antioxidant defenses and buffering capacity against inflammatory cytokine storms [41]. This effect is particularly pronounced in the setting of lymphocytopenia and subsequent immune dysregulation. The resulting systemic vulnerability predisposes patients to severe microvascular damage during ischemia–reperfusion and maladaptive cardiac remodeling, ultimately leading to adverse clinical outcomes.

Of note, our analysis of subgroups identified a significant interaction between the HALP score and age in relation to 365 days mortality. The protective effect of a high HALP score was markedly stronger in patients aged ≥70 years, but was not significant in the younger cohort. A likely explanation for this is that elderly patients, because of their diminished physiological reserves, are more vulnerable to the nutritional and immune insults captured by the HALP score. In contrast, mortality in younger patients may be driven by more aggressive pathophysiological factors that overshadow the HALP parameters. This finding highlights the utility of the HALP score as a particularly crucial long-term prognostic marker for risk-stratification of elderly, critically ill AMI patients.

In summary, a low HALP score reflects an impaired immune defense and poor nutritional reserves, resulting in uncontrolled inflammatory responses and oxidative stress. This weakened immune state further increases the patients’ susceptibility to complications such as infections, while hindering myocardial repair, delaying functional recovery, and ultimately worsening patient prognosis. Conversely, when the HALP score exceeds a certain threshold, it indicates a relatively favorable immune and nutritional status. Beyond this point, further increases in HALP score provide diminishing marginal benefit in terms of prognosis, representing a plateau in its predictive utility. Moreover, recent findings indicate that certain glucose-lowering drugs, such as GLP-1 receptor agonists and SGLT2 inhibitors, may provide cardioprotective effects in AMI, irrespective of their glycemic control function. These agents exhibit anti-inflammatory and endothelial-stabilizing properties, which may interact with nutritional and inflammatory pathways reflected in the HALP score [42]. Although not addressed in the present study, this evolving therapeutic landscape warrants further investigation. Compared to previous studies, our work offers several novel insights. Pannu [43] emphasized the theoretical advantages of HALP and CALLY as systemic indices to supplement traditional ACS risk models, but provided no primary data from critically ill cohorts. Yılmaz *et al*. [44] focused on elderly AMI patients (≥75 years) undergoing PCI and identified HALP as a long-term predictor of mortality in a relatively small, elective cohort. In contrast, our study targeted critically ill AMI patients in the ICU setting and provided robust statistical modeling, including nonlinear and machine learning analyses. We identified a clinically actionable threshold (HALP = 19.41) to facilitate early risk stratification and personalized care.

Nonetheless, this study has several limitations that should be taken into consideration. First, a key limitation is the selection bias associated with the exclusion of 73.8% of patients who lacked HALP data. Because the patients included in the study cohort were more unwell and had higher mortality, our findings on the prognostic value of the HALP score apply mainly to this high-risk group and may not generalize to less severe AMI populations. Second, due to a substantial amount of missing data for inflammatory biomarkers and lipid profiles in the database, these variables could not be incorporated into our analysis. This absence may limit a more comprehensive understanding of the immune and metabolic pathways involved in AMI, potentially affecting the completeness of our predictive models. Third, the retrospective and single-center design of the study is a further limitation, with the findings being susceptible to selection bias and residual confounding, thus restricting our ability to establish causality. Fourth, this was a retrospective, single-center study based on a U.S. tertiary academic hospital cohort, with patients limited to those admitted to the ICU for AMI. Caution is warranted when extrapolating the findings to AMI populations outside the ICU, in resource-limited settings, or in different healthcare systems. Furthermore, the findings may not fully apply to patients with specific clinical subtypes, such as ST-elevation or non-ST-elevation MI. Lastly, the HALP score in this study was calculated from lab values obtained during the initial 24 h of the patient’s stay in ICU. This may reflect acute physiological stress rather than chronic nutritional or inflammatory status. Therefore, caution is warranted when interpreting HALP as a modifiable biomarker. To overcome these shortcomings, future investigations should focus on large-scale, prospective studies conducted at multiple centers. Such a design would be instrumental in validating our findings across more heterogeneous AMI populations, including non-ICU patients and those from different healthcare systems. This would enhance the generalizability of our findings and reduce selection bias. Furthermore, additional studies should aim for the systematic collection of serial HALP measurements alongside a comprehensive panel of inflammatory and metabolic biomarkers. Such an approach is crucial for mitigating confounding factors and better elucidating the temporal dynamics and causal role of the HALP score in AMI prognosis.

## 5. Conclusions

The present study validates the HALP score as an independent predictor of mortality for patients with AMI. Moreover, machine learning models incorporating the HALP score showed strong performance in predicting mortality risk, further highlighting its potential utility in clinical decision-making. These results support use of the HALP score as a practical, economical, and objective tool for early risk stratification and outcome prediction in critically ill patients with AMI.

## Data Availability

This study analyzed publicly available datasets from the MIMIC-IV v3.1 database (http://physionet.org/content/mimiciv/3.1/).

## Abbreviations

HALP, hemoglobin, albumin, lymphocyte, and platelet; AMI, acute myocardial infarction; ICU, intensive care unit; MIMIC, Medical Information Mart for Intensive Care; AUC, area under the receiver operating characteristic curve; RCS, restricted cubic spline; HR, hazard ratio; CI, confidence interval; RR, respiratory rate; SBP, systolic blood pressure; DBP, diastolic blood pressure; SPO_2_, peripheral capillary oxygen saturation; Hb, hemoglobin; INR, international normalized ratio; PH, potential of hydrogen; PTT, partial thromboplastin time; WBC, white blood cell count; PCO_2_, partial pressure of carbon dioxide; Cr, creatinine; BUN, blood urea nitrogen; GLU, glucose; PO_2_, partial pressure of oxygen; AF, atrial fibrillation; CA, cancer; CKD, chronic kidney disease; CHF, congestive heart failure; COPD, chronic obstructive pulmonary disease; MV, mechanical ventilation; APS-III, Acute Physiology Score III; CCI, Charlson Comorbidity Index; GCS, Glasgow Coma Scale; SAPS-II, Simplified Acute Physiology Score II; SOFA, Sequential Organ Failure Assessment; SII, Systemic Immune-Inflammation Index; SIRI, Systemic Inflammatory Response Index; NLR, neutrophil-to-lymphocyte ratio; PNI, Prognostic Nutritional Index; CONUT, Controlling Nutritional Status; DAMPs, damage-associated molecular patterns; ROS, reactive oxygen species; IL-6, interleukin-6; LASSO, least absolute shrinkage and selection operator; ENet, elastic net; LightGBM, Light Gradient Boosting Machine; MLP, multilayer perceptron; RF, random forest; XGBoost, extreme gradient boosting; ROC, receiver operating characteristic; DCA, decision curve analysis; VIF, variance inflation factor.

## Supplementary Material

Supplementary material associated with this article can be found, in the online version, at https://doi.org/10.31083/RCM43942.
