# Supplementary material for: Association Between the Hemoglobin, Albumin, Lymphocyte, and Platelet (HALP) Score and Adverse Outcomes in Critically Ill Patients With Acute Myocardial Infarction: A Retrospective Study and Machine Learning Analysis
Source: Rev Cardiovasc Med. 2025 Aug 20;26(8):43942. doi: 10.31083/RCM43942 (PMC12415744; doi:10.31083/RCM43942)

**Supplementary materials**

**Supplementary Tables**

**Supplementary Table 1** All Variables extracted from the MIMIC-IV Database.

| **Category** | **Variables Included** |
| --- | --- |
| Demographics | Age, Gender, and Race. |
| Vital parameters | Heart rate, RR, SBP, DBP, and Spo2. |
| Laboratory indicators | Hemoglobin, Albumin, Lymphocyte count, Platelet count, INR, pH, PTT, WBC, PaCO₂, Creatinine, Potassium, Sodium, BUN, Lactate, Glucose, PaO₂, Anion gap, and Neutrophils. |
| Underlying comorbid conditions | AF, CA, CKD, CHF, COPD, Diabetes, Hypertension, Sepsis, and Stroke. |
| Medication usage | Aspirin, Beta-blockers, Clopidogrel, and Statin. |
| Clinical interventions | CRRT, Invasive MV, and Noninvasive MV. |
| Severity scores | APS - III, CCI, GCS, SAPS - II, and SOFA. |
| Clinical outcomes | 90-day hospital Mortality and 365-day hospital Mortality. |
| RR, respiratory rate; SBP, systolic blood pressure; DBP, diastolic blood pressure; SpO2, peripheral capillary oxygen saturation; Hb, hemoglobin; INR, international normalized ratio; PH, potential of hydrogen; PTT, partial thromboplastin time; WBC, white blood cell; PcO2, partial pressure of carbon dioxide; Cr, creatinine; BUN, blood urea nitrogen; GLU, glucose; PO2, partial pressure of oxygen; AF, atrial fibrillation; CA, cancer; CKD, chronic kidney disease; CHF, congestive heart failure; COPD, chronic obstructive pulmonary disease; CRRT, continuous renal replacement therapy; MV, mechanical ventilation; APSIII, acute physiology score III; CCI, Charlson comorbidity index; GCS, Glasgow coma scale; SAPSII, simplified acute physiology score II; SOFA, sequential organ failure assessment. | |

**Supplementary Table 2** Variance inflation factor analysis for multicollinearity assessment among clinical covariates.

| **Characteristic** | **GVIF** | **Df** | **GVIF^(1/(2*Df))** |
| --- | --- | --- | --- |
| Age | 1.788673 | 1 | 1.337413 |
| Gender | 1.280781 | 1 | 1.131716 |
| Race | 1.528674 | 2 | 1.111933 |
| Heart rate | 1.482330 | 1 | 1.217510 |
| RR | 1.304218 | 1 | 1.142024 |
| SBP | 1.929657 | 1 | 1.389121 |
| DBP | 1.729455 | 1 | 1.315087 |
| SpO2 | 1.341751 | 1 | 1.158340 |
| INR | 1.485801 | 1 | 1.218934 |
| PH | 2.519220 | 1 | 1.587205 |
| PTT | 1.256235 | 1 | 1.120819 |
| WBC | 1.182494 | 1 | 1.087425 |
| PcO2 | 1.939191 | 1 | 1.392549 |
| CR | 2.572417 | 1 | 1.603876 |
| Potassium | 1.427321 | 1 | 1.194705 |
| Sodium | 1.513638 | 1 | 1.230300 |
| BUN | 2.437010 | 1 | 1.561093 |
| Lactate | 3.832792 | 1 | 1.957752 |
| GLU | 1.464142 | 1 | 1.210017 |
| PO2 | 1.396753 | 1 | 1.181843 |
| Anion gap | 3.486452 | 1 | 1.867204 |
| AF | 1.416757 | 1 | 1.190276 |
| CA | 1.257484 | 1 | 1.121376 |
| CKD | 1.780476 | 1 | 1.334345 |
| CHF | 1.341339 | 1 | 1.158162 |
| COPD | 1.231220 | 1 | 1.109604 |
| Diabetes | 1.553770 | 1 | 1.246503 |
| Hypertension | 1.505267 | 1 | 1.226893 |
| Sepsis | 1.268984 | 1 | 1.126492 |
| Stroke | 1.198734 | 1 | 1.094867 |
| Aspirin | 1.700748 | 1 | 1.304127 |
| Beta-blockers | 1.356738 | 1 | 1.164791 |
| Clopidogrel | 1.223716 | 1 | 1.106217 |
| Statin | 1.559308 | 1 | 1.248723 |
| Invasive MV | 1.772578 | 1 | 1.331382 |
| Noninvasive MV | 1.056992 | 1 | 1.028101 |
| APS - III | 3.742584 | 1 | 1.934576 |
| CCI | 2.089707 | 1 | 1.445582 |
| SAPS - II | 1.783116 | 1 | 1.335334 |
| SOFA | 3.887130 | 1 | 1.971581 |
| GVIF, generalized variance inflation factor; Df, degrees of freedom; RR, respiratory rate; SBP, systolic blood pressure; DBP, diastolic blood pressure; SpO2, peripheral capillary oxygen saturation; Hb, hemoglobin; INR, international normalized ratio; PH, potential of hydrogen; PTT, partial thromboplastin time; WBC, white blood cell; PcO2, partial pressure of carbon dioxide; Cr, creatinine; BUN, blood urea nitrogen; GLU, glucose; PO2, partial pressure of oxygen; AF, atrial fibrillation; CA, cancer; CKD, chronic kidney disease; CHF, congestive heart failure; COPD, chronic obstructive pulmonary disease; CRRT, continuous renal replacement therapy; MV, mechanical ventilation; APSIII, acute physiology score III; CCI, Charlson comorbidity index; GCS, Glasgow coma scale; SAPSII, simplified acute physiology score II; SOFA, sequential organ failure assessment. | | | |

**Supplementary Table 3** Hyperparameter tuning ranges and final selected values for machine learning models

| **Model** | **Parameter** | **Tuning Range** | **Final Value** |
| --- | --- | --- | --- |
| **Decision Tree** | cost_complexity | 1×10⁻¹⁰ – 0.1 | 0.00001 |
|  | tree_depth | 1 – 15 | 11 |
|  | min_n | 2 – 40 | 40 |
| **Random Forest** | mtry | 2 – 10 | 6 |
|  | trees | 100 – 1000 | 800 |
|  | min_n | 7 – 55 | 55 |
| **XGBoost** | mtry | 2 – 6 | 3 |
|  | min_n | 2 – 40 | 23 |
|  | tree_depth | 1 – 15 | 14 |
|  | learn_rate | 0.001 – 0.3 | 0.0313 |
|  | loss_reduction | 1×10⁻¹⁰ – 0.1 | 1.69E-10 |
|  | sample_size | 0.2 – 1 | 0.753 |
|  | trees | 1000 (fixed) | 1000 |
| **LightGBM** | mtry | 2 – 10 | 6 |
|  | trees | 1 – 2000 | 1111 |
|  | min_n | 2 – 40 | 40 |
|  | tree_depth | 1 – 15 | 7 |
|  | learn_rate | 1e-10 – 0.3 | 1.00E-10 |
|  | loss_reduction | 1e-10 – 1 | 3.59E-08 |
| **SVM** | cost | 0.0001 – 10 | 0.0011 |
|  | rbf_sigma | 1e-7 – 1 | 2.96E-05 |
| **MLP** | hidden_units | 1 – 10 | 7 |
|  | penalty | 1e-8 – 1 | 1 |
|  | epochs | 10 – 1000 | 340 |
| **KNN** | neighbors | 5 – 35 | 35 |
|  | weight_func | rectangular / triangular | triangular |
| **Ridge** | penalty | 1e-10 – 1 | 0.997 |
|  | mixture | 0 (fixed, Ridge) | 0 |
| **Elastic Net** | penalty | 1e-10 – 1 | 0.378 |
|  | mixture | 0 – 1 | 0.0969 |
| XGBoost, Extreme Gradient Boosting; LightGBM, Light Gradient Boosting Machine; SVM, Support Vector Machine; MLP, Multilayer Perceptron; KNN, k-Nearest Neighbors; Ridge, Ridge Regression. | | | |

**Supplementary Table 4** Baseline characteristics of included and excluded patients due to missing HALP score parameters

| **Characteristic** | **Overall (n = 7,525)** | **Excluded (n = 6,707)** | **Included (n = 818)** | **p-value** |
| --- | --- | --- | --- | --- |
| Age (year) | 72(62,81) | 72(62,81) | 71(62,80) | 0.079 |
| Gender, n (%) |  |  |  | 0.257 |
| Female | 2,753(37%) | 2,439(36%) | 314(38%) |  |
| Male | 4,772(63%) | 4,268(64%) | 504(62%) |  |
| Race, n (%) |  |  |  | <0.001 |
| Black | 678(9.0%) | 607(9.1%) | 71(8.7%) |  |
| White | 4,834(64%) | 4,359(65%) | 475(58%) |  |
| Others | 2,013(27%) | 1,741(26%) | 272(33%) |  |
| Heart rate (bpm) | 83(74,96) | 83(73,96) | 87(75,101) | <0.001 |
| RR (bpm) | 18(15,22) | 18(14,22) | 20(16,25) | <0.001 |
| SBP (mmHg) | 119(104,137) | 119(105,137) | 119(104,138) | 0.865 |
| DBP (mmHg) | 66(56,79) | 66(56,79) | 69(59,81) | <0.001 |
| SpO2 (%) | 98(95,100) | 98(96,100) | 97(94,100) | <0.001 |
| Hemoglobin (g/dL) | 10.40(8.80,12.10) | 10.40(8.80,12.10) | 10.65(8.80,12.70) | 0.011 |
| Albumin (g/dL) | 3.30(2.80,3.70) | 3.30(2.90,3.70) | 3.20(2.80,3.60) | <0.001 |
| Lymph (10^9^/L) | 1.44(0.84,2.22) | 1.68(1.06,2.46) | 1.07(0.65,1.70) | <0.001 |
| Platelet (10^9^/L) | 188(139,248) | 187(139,247) | 198(141,255) | 0.092 |
| INR | 1.30(1.10,1.50) | 1.30(1.10,1.50) | 1.30(1.10,1.50) | 0.556 |
| PH | 7.37(7.32,7.42) | 7.38(7.32,7.42) | 7.35(7.28,7.41) | <0.001 |
| PTT (S) | 33(28,45) | 33(28,44) | 34(28,51) | 0.030 |
| WBC (10^9^/L) | 11.5(8.4,15.5) | 11.4(8.4,15.3) | 12.5(8.9,17.3) | <0.001 |
| PcO2 (mmHg) | 41(36,46) | 41(36,46) | 41(35,48) | 0.588 |
| Cr (mg/dL) | 1.10(0.80,1.70) | 1.10(0.80,1.70) | 1.30(0.90,2.10) | <0.001 |
| Potassium (mmol/L) | 4.20(3.90,4.70) | 4.20(3.90,4.70) | 4.30(3.90,4.70) | 0.313 |
| Sodium (mmol/L) | 138(136,141) | 138(136,141) | 138(136,141) | 0.378 |
| BUN (mg/dL) | 22(15,37) | 22(15,35) | 26(16,46) | <0.001 |
| Lactate (mmol/L) | 1.90(1.30,2.70) | 1.90(1.30,2.70) | 1.90(1.30,3.10) | 0.011 |
| GLU (mg/dL) | 132(109,177) | 131(109,174) | 147(113,207) | <0.001 |
| PO2 (mmHg) | 129(59,304) | 153(66,315) | 65(41,121) | <0.001 |
| Anion gap (mmol/L) | 14(11,17) | 14(11,17) | 15(13,18) | <0.001 |
| Neuts (10^9^/L) | 9.8(6.9,13.7) | 9.6(6.9,13.0) | 10.4(6.9,14.9) | 0.001 |
| AF, n (%) | 3,373(45%) | 3,005(45%) | 368(45%) | 0.920 |
| CA, n (%) | 1,660(22%) | 1,493(22%) | 167(20%) | 0.230 |
| CKD, n (%) | 3,070(41%) | 2,750(41%) | 320(39%) | 0.301 |
| CHF, n (%) | 4,525(60%) | 4,019(60%) | 506(62%) | 0.286 |
| COPD, n (%) | 1,748(23%) | 1,568(23%) | 180(22%) | 0.380 |
| Diabetes, n (%) | 3,634(48%) | 3,274(49%) | 360(44%) | 0.009 |
| Hypertension, n (%) | 4,385(58%) | 4,015(60%) | 370(45%) | <0.001 |
| Sepsis, n (%) | 4,459(59%) | 3,863(58%) | 596(73%) | <0.001 |
| Stroke, n (%) | 1,330(18%) | 1,204(18%) | 126(15%) | 0.071 |
| Aspirin, n (%) | 4,810(64%) | 4,271(64%) | 539(66%) | 0.213 |
| Beta-blockers, n (%) | 5,182(69%) | 4,701(70%) | 481(59%) | <0.001 |
| Clopidogrel, n (%) | 1,530(20%) | 1,374(20%) | 156(19%) | 0.342 |
| Statin, n (%) | 4,913(65%) | 4,373(65%) | 540(66%) | 0.644 |
| CRRT, n (%) | 312(4.1%) | 234(3.5%) | 78(9.5%) | <0.001 |
| Invasive MV, n (%) | 3,412(45%) | 3,016(45%) | 396(48%) | 0.062 |
| Noninvasive MV, n (%) | 260(3.5%) | 239(3.6%) | 21(2.6%) | 0.141 |
| APS - III | 42(31,55) | 41(31,54) | 48(35,65) | <0.001 |
| CCI | 6(4,8) | 6(4,8) | 6(5,9) | <0.001 |
| GCS | 15(14,15) | 15(14,15) | 15(14,15) | 0.027 |
| SAPS - II | 37(30,46) | 37(30,46) | 41(31,52) | <0.001 |
| SOFA | 5(2,7) | 4(2,7) | 6(3,10) | <0.001 |
| 90-day Mortality, n (%) | 1,470(20%) | 1,220(18%) | 250(31%) | <0.001 |
| 365-day Mortality, n (%) | 2,085(28%) | 1,755(26%) | 330(40%) | <0.001 |
| HALP, Hemoglobin, Albumin, Lymphocyte, and Platelet; RR, respiratory rate; SBP, systolic blood pressure; DBP, diastolic blood pressure; SpO2, peripheral capillary oxygen saturation; Hb, hemoglobin; INR, international normalized ratio; PH, potential of hydrogen; PTT, partial thromboplastin time; WBC, white blood cell; PcO2, partial pressure of carbon dioxide; Cr, creatinine; BUN, blood urea nitrogen; GLU, glucose; PO2, partial pressure of oxygen; AF, atrial fibrillation; CA, cancer; CKD, chronic kidney disease; CHF, congestive heart failure; COPD, chronic obstructive pulmonary disease; CRRT, continuous renal replacement therapy; MV, mechanical ventilation; APSIII, acute physiology score III; CCI, Charlson comorbidity index; GCS, Glasgow coma scale; SAPSII, simplified acute physiology score II; SOFA, sequential organ failure assessment. | | | | |

**Supplementary Table 5** Relative contribution of HALP components to 90-day mortality (dominance analysis)

| **HALP Component** | **Relative Contribution (%)** |
| --- | --- |
| Albumin | 77.80% |
| Hemoglobin | 13.50% |
| Platelet | 7.90% |
| Lymphocytes | 0.80% |
| HALP, Hemoglobin, Albumin, Lymphocyte, and Platelet. | |

**Supplementary Table 6** Interaction effects between HALP components in predicting 90-day mortality (cox proportional hazards model)

| **Interaction Term** | **HR** | **95% CI** | ***P* value** |
| --- | --- | --- | --- |
| Albumin × Hemoglobin | 1.027 | 0.940–1.123 | 0.55 |
| Albumin × Lymphocytes | 0.995 | 0.878–1.127 | 0.935 |
| Albumin × Platelet | 1.001 | 0.999–1.004 | 0.19 |
| Hemoglobin × Lymphocytes | 0.992 | 0.977–1.007 | 0.307 |
| Hemoglobin × Platelet | 1 | 1.000–1.001 | 0.604 |
| Lymphocytes × Platelet | 1 | 0.999–1.000 | 0.347 |
| HALP, Hemoglobin, Albumin, Lymphocyte, and Platelet; HR, hazard ratio; CI, confidence interval. | | | |

**Supplementary Table 7** Comparison of different models in predicting the 90-day mortality of patients with acute myocardial infarction admitted to the intensive care unit.

|  | **Predictive Model** | **AUROC** | **IDI** | ***P* value** | **NRI** | ***P* value** |
| --- | --- | --- | --- | --- | --- | --- |
| Training set | ENet | 0.774 |  |  |  |  |
|  | CCI | 0.672 | 0.066 | <0.001 | 0.291 | <0.001 |
|  | GCS | 0.558 | 0.133 | <0.001 | 0.752 | <0.001 |
| Validation set | ENet | 0.78 |  |  |  |  |
|  | CCI | 0.674 | 0.079 | 0.005 | 0.445 | <0.001 |
|  | GCS | 0.54 | 0.156 | <0.001 | 0.924 | <0.001 |
| Enet, elastic net regression; CCI, Charlson comorbidity index; GCS, Glasgow coma scale; AUROC, area under the receiver operating characteristic curve; IDI, integrated discrimination improvement; NRI, net reclassification improvement. | | | | | | |

**Supplementary Figures**

**Supplementary Fig. 1** Decision Curve Analysis (DCA) comparing the net benefit of the ENet model against charlson comorbidity index (CCI) and glasgow coma scale (GCS) for predicting 90-day mortality.

**
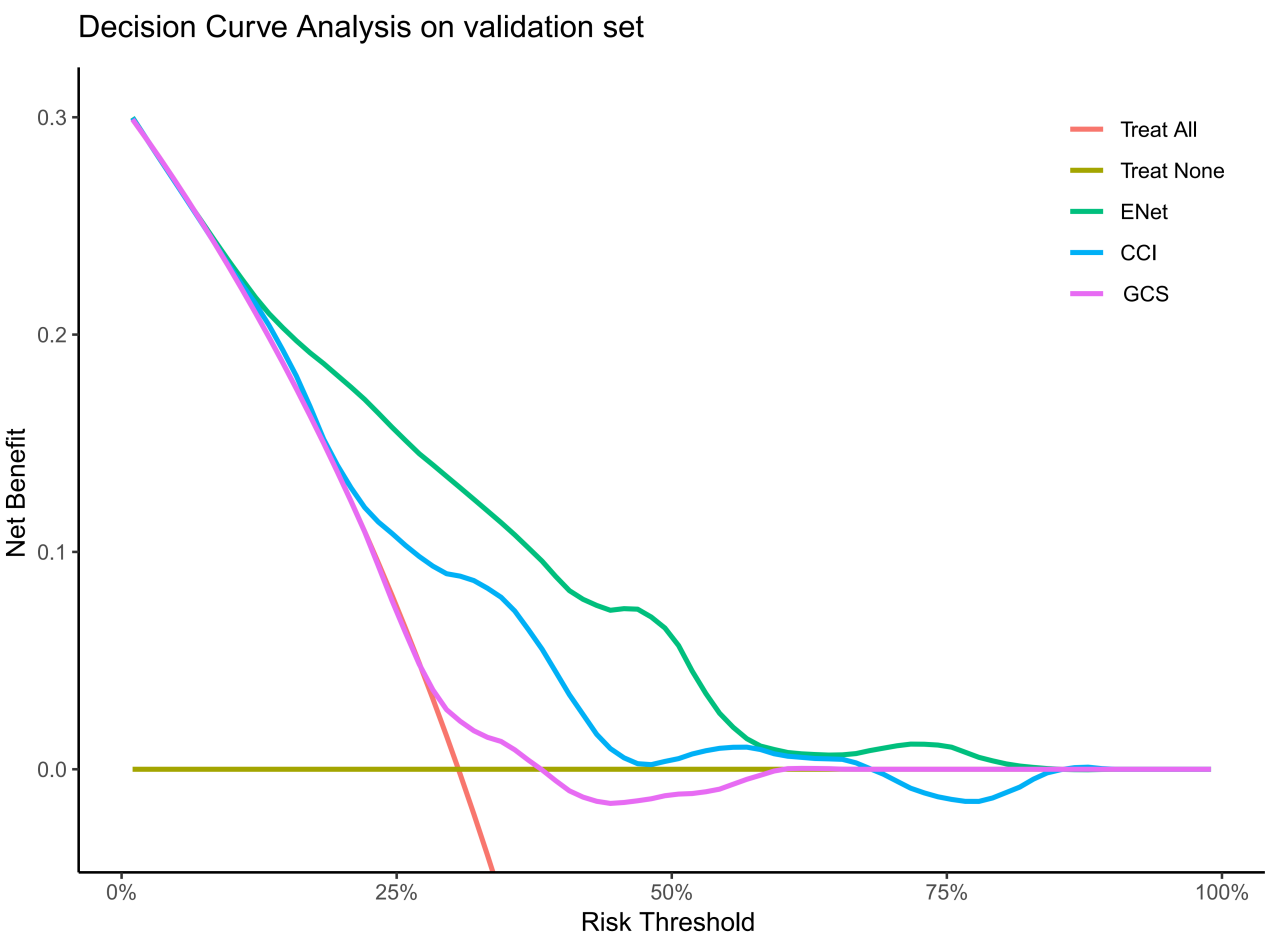
**

**Supplementary Fig. 2** SHAP summary plot showing the impact of each feature on model predictions.

**
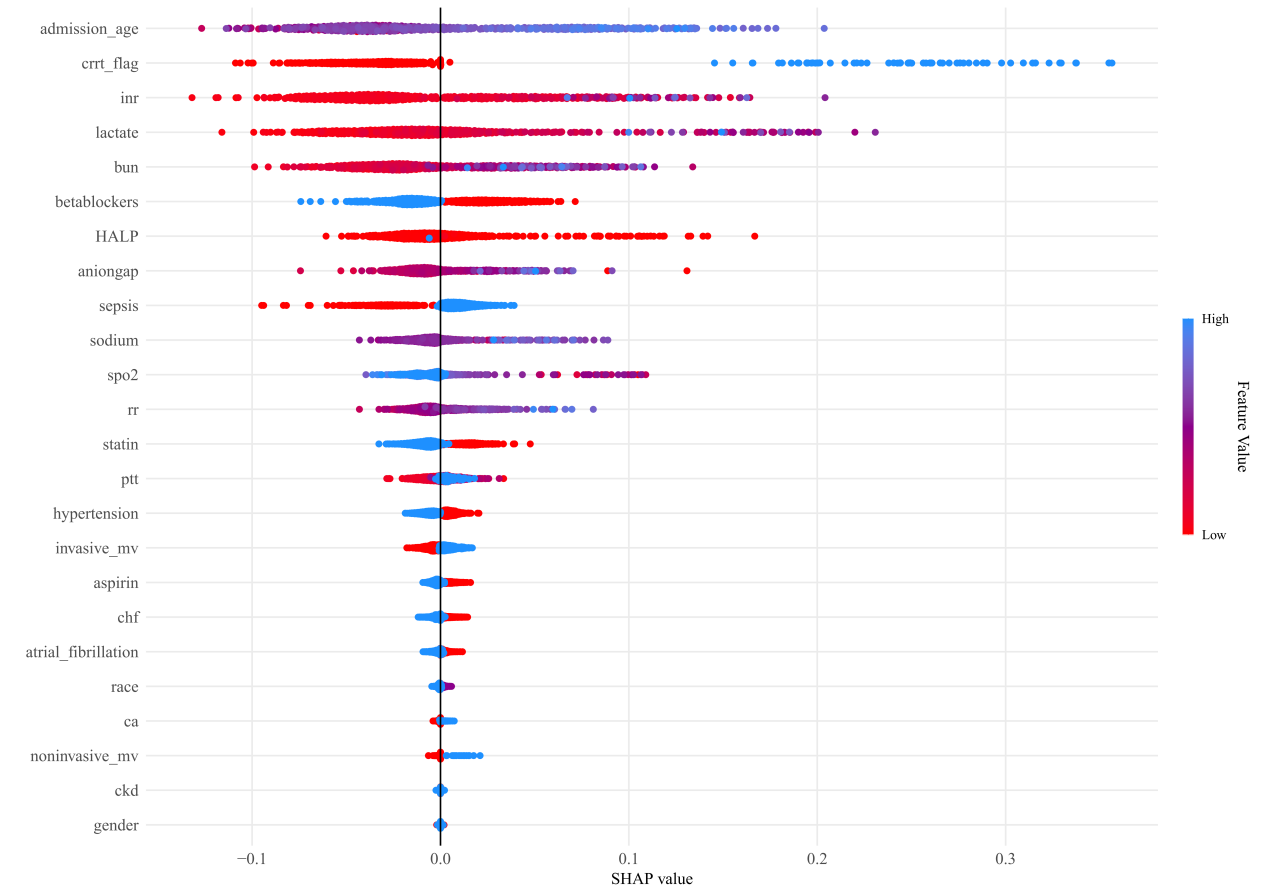
**

**Supplementary Fig. 3** Mean absolute SHAP values ranking the global importance of each feature.


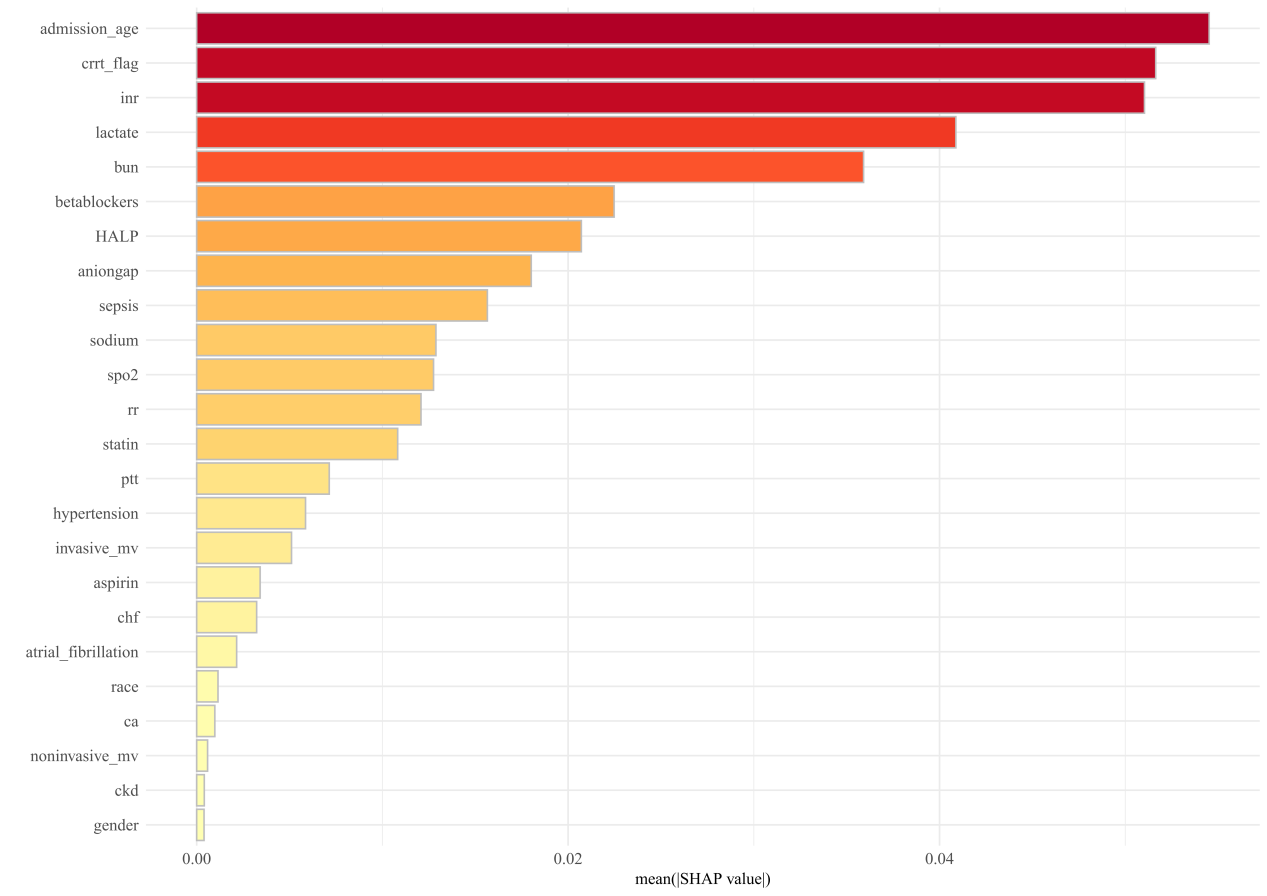

Supplement: Supplementary file 1 [file 2153-8174-26-8-43942-s1.docx]
